# Prevalence and Molecular Characterisation of *Blastocystis* sp. Infecting Free-Ranging Primates in Colombia

**DOI:** 10.3390/pathogens12040569

**Published:** 2023-04-06

**Authors:** Silvia Rondón, Serena Cavallero, Andrés Link, Camila González, Stefano D’Amelio

**Affiliations:** 1 Department of Public Health and Infectious Diseases, Sapienza University of Rome, Piazzale Aldo Moro 5, 00185 Rome, Italy; 2 Laboratorio de Ecología de Bosques Tropicales y Primatología (LEBTYP), Departamento de Ciencias Biológicas, Universidad de los Andes, Cra. 1 N° 18a-12, Bogotá 111711, Colombia; 3 Centro de Investigaciones en Microbiología y Parasitología Tropical (CIMPAT), Departamento de Ciencias Biológicas, Universidad de los Andes, Cra. 1 N° 18a-12, Bogotá 111711, Colombia

**Keywords:** *Blastocystis* sp., free-ranging primates, Colombia, molecular characterisation

## Abstract

Infection with *Blastocystis* sp. has been reported in free-living and captive non-human primates (NHPs); however, surveys on *Blastocystis* sp. from north-western South America are scarce. This study aimed to identify *Blastocystis* sp. in free-ranging NHPs living in Colombia. A total of 212 faecal samples were collected from *Ateles hybridus*, *Cebus versicolor*, *Alouatta seniculus*, *Aotus griseimembra*, *Sapajus apella*, and *Saimiri cassiquiarensis*. Smears and flotation were used for morphological identification. For samples microscopically classified as positive for *Blastocystis* sp., we used conventional PCR to amplify and sequence two regions of the SSU rRNA gene and used Maximum Likelihood methods and Median Joining Network analyses for phylogenetic analyses. Via microscopy, 64 samples were *Blastocystis* sp. positive. Through molecular analyses, 18 sequences of *Blastocystis* sp. subtype 8 (ST8) were obtained. Strain and allele assignment together with a comparative phylogenetic approach confirmed that the sequences were ST8. Alleles 21, 156, and 157 were detected. Median Joining network analyses showed one highly frequent haplotype shared by specimens from Colombia and Peru and close relationships between haplotypes circulating in NHPs from Colombia, Ecuador, Brazil, and Mexico. This survey could support the elaboration of a more accurate epidemiological picture of the *Blastocystis* sp. infecting NHPs.

## 1. Introduction

Non-human primates (NHPs) have been found infected with a diverse array of intestinal parasites, including many protozoans and protists. *Blastocystis* is one of the most widespread enteric protists infecting animals such as reptiles, birds, and mammals (including humans). Its high occurrence in animal and human caeca and large intestine has raised a debate regarding its pathogenic role [1], as is it frequently found in asymptomatic individuals [2]. However, *Blastocystis* sp. may cause clinical signs including abdominal pain, constipation, and flatulence with diarrhoea [3]. *Blastocystis* has been found infecting NHPs, both free-living and captive platyrrhines and catarrhines, in areas where different subtypes (STs) have been identified: ST1-5, ST8, ST13, ST15, and ST39 [4,5,6,7]. Detailed information about *Blastocystis* sp. prevalence and STs occurrence in free-ranging and captive primates around the world has been recently reviewed by Hublin et al. [2].

In NHPs native to Central America and South America, studies on *Blastocystis* sp. have been mainly conducted on captive individuals and resulted in the identification of the following strains: ST1 in *Lagothrix* sp. in the United Kingdom [8], *Aotus* sp. in Brazil [5], *Leontopithecus chrysomelas* and *Pithecia pithecia* in France [4,9], and *Ateles paniscus* and *Saguinus labiatus* in the Netherlands [4]. ST2 was reported in *Alouatta seniculus*, *Ateles fusciceps*, and *Ateles belzebuth* in Brazil [6]; *Pithecia pithecia* and *Ateles hybridus* in France [4,9]; and *Lagothrix* sp. in the United Kingdom. ST3 was reported in *A. seniculus* in Brazil [6], *Callithrix jacchus* and *Lagothrix lagotricha* in the United Kingdom [10], and *S*. *labiatus* in the Netherlands [4], while ST8 was reported in *Alouatta caraya* in Germany and the United Kingdom [4]; *Alouatta* sp., *Ateles* sp., *Ateles fusciceps*, *L. lagotricha,* and *Aotus* sp. in Brazil [5,6]; and *Lagothrix* sp. in the United Kingdom [8].

In free-ranging NHPs, ST4 has been identified infecting *Alouatta* sp. in Colombia [11], ST8 in *Alouatta palliata aequatorialis* in Ecuador [12], *A. palliata* and *Alouatta pigra* in Mexico [13], *Aotus nigriceps* in Peru [14], and *A. caraya* in Brazil [6], while ST1–ST2 have been reported infecting *A. palliata* and *Alouatta pigra* in Mexico [13]. Overall, ST4 has been described in free-ranging platyrrhines and ST3 has been reported in captive ones, while ST1, ST2, and ST8 have been reported in both free-ranging and captive platyrrhines.

Moreover, infections with *Blastocystis* were found via PCR screening in captive *A. caraya*, *Alouatta fusca*, *Callithrix argentata*, *C. jacchus*, *Sapajus apella*, and *L. chrysomelas* in Brazil, without subtype characterisation [6]. Likewise, some other studies based on morphology have identified *Blastocystis* infection in *A. seniculus*, *A. pigra*, *A. caraya*, *Saimiri sciureus*, *S. apella*, *A. belzebuth*, and *Aotus azarae* in Argentina, Mexico, and Peru [15,16,17,18,19].

In addition to records of *Blastocystis* ST4 infecting red howler monkeys in Colombia [11], other subtypes have been found in the country, including ST1-3, ST5, ST10, ST14, ST21, ST23-26, and ST32-34 infecting domestic animals (e.g., cows, dogs, goats, horses, pigs, sheep, and rabbits) in Cundinamarca, Casanare, Boyacá, and Santander Departments, among others [20,21]; ST6 infecting birds; and ST8 infecting the common opossum (*Didelphis marsupialis*) [11]. In humans, ST1–4, ST6, ST7, and ST16 [11,22,23] have been reported in Colombia, while the circulation of additional STs (e.g., ST5, ST8, ST9, and ST24) has been confirmed in other countries in North and South America [3]. Additionally, ST28–30 and ST35–37 have been recently reported in the Americas [21,24].

As surveys on *Blastocystis* sp. infecting NHPs from Colombia are still scarce, this study aimed to increase the knowledge on the molecular epidemiology of *Blastocystis* sp. infecting free-ranging primates living in fragmented forests in Colombia.

## 2. Materials and Methods

### 2.1. Sampling

Fieldwork was carried out between December 2019 and February 2022 in seven locations in Colombia: San Juan in Santander Department; Cumaral, Cabuyaro, Guacavía, and Villavicencio in Meta Department; and Maní and Yopal in Casanare Department. Primates were followed from dawn to dusk, and one faecal sample per individual was collected from the soil immediately after defecation. For each sample, one aliquot was stored in 10% formalin solution, and another aliquot was stored in 96% ethanol solution. Overall, 212 samples were collected from free-ranging *Ateles hybridus*, *Cebus versicolor*, *Alouatta seniculus*, *Aotus griseimembra*, *Sapajus apella*, and *Saimiri cassiquiarensis.*

### 2.2. Microscopy Analyses

Samples stored in 10% formalin solution were used to perform smears with 1% iodine solution and 0.85% saline solution [25]. For each faecal sample, two microscope slides were mounted and systematically examined under a microscope using magnifications at 100×, 400×, and 1000×. Additionally, flotation with a salt–sugar solution was carried out. Each sample was placed in a 15 mL Falcon tubes with flotation solution and then centrifuged at 220 RCF for 5 min. Flotation solution was added until a slight positive meniscus was formed; then, a coverslip was placed on the top of the tube. After 10 min, the coverslip was removed and placed into a microscope slide. Thereafter, the meniscus was taken and placed into a new 15 mL Falcon tube, and flotation solution was added until the formation of a slight positive meniscus. A coverslip was placed on the top of the tube; after 10 min, the coverslip was removed and placed into a microscope slide with a drop of iodine solution. For each faecal sample, one microscope slide was examined using magnifications at 100×, 400×, and 1000× after the flotation procedure.

### 2.3. Molecular Analyses

For samples microscopically classified as positive for *Blastocystis* sp., the respective aliquots stored in 96% ethanol solution were individually subjected to DNA extraction using the Isolate II Fecal DNA Kit (Meridian Bioscience, London, UK) according to the manufacturer’s protocol. Two conventional PCRs were performed using the pair of primers BhRDr-RD5 and Blast505-532-Blast998-1017 in order to amplify two different regions of the small subunit ribosomal RNA (SSU rRNA or 18S) gene according to published methods [8,26]. All PCR products were visualized on a 1% agarose gel stained with SYBR Safe, and positive samples were purified using Sure Clean Plus (Bioline, London, UK) and shipped to an external company for bidirectional sequencing (Eurofins Genomics). Sequences were manually edited using Trace implemented in MEGA7 [27] to infer consensus sequences by retaining only high-quality electropherograms and then checked for multiple peaks to rule out potential mixed infections. Thereafter, consensus sequences were used as input for BLAST search and strain/allele assignment using the PubMLST.org website. Two datasets named according to primers used (Scicluna and Santin) were obtained and used for the following analyses.

Alignments of sequences were carried out per each partial region of the SSU rRNA gene studied using ClustalW in MEGA7. Initial comparisons with the aim of inferring the strain identity and phylogenetic relationships were performed, including reference sequences of all available strains and a proper outgroup (Supplementary Table S1). The best evolutionary model was obtained using ModelTest implemented in MEGA7, and the Maximum Likelihood (ML) method was used to infer phylogeny with the aim of confirming relationships between the *Blastocystis* sequences identified herein and the other strains, for which statistical support at nodes was provided according to bootstrap. Moreover, evolutionary relationships between sequences belonging to the same strain circulating in NHPs in Mexico and South America (Table 1) were explored using Median Joining Network analyses [28] carried out with PopART [29].

### 2.4. Data Analyses

Quantitative Parasitology Software was used to calculate the prevalence of *Blastocystis* per primate species and study site with 95% confidence intervals.

## 3. Results

### 3.1. Microscopy Analyses

Sixty-four samples (30.2%) were classified as positive for *Blastocystis* infecting NHPs from five of the seven study locations and including all NHP species except *A. griseimembra* (Table 2). The prevalence of *Blastocystis* according to NHP species was 53.8% [25.1–80.8%] for *A. hybridus*, 30.0% [11.9–54.3%] for *C. versicolor*, 60.7% [46.8–73.5%] for *A. seniculus*, 28.6% [11.3–52.2%] for *S. apella*, and 11.3% [5.8–19.4%] for *S. cassiquiarensis*.

### 3.2. Molecular Analyses

Overall, we obtained ten high-quality sequences from the PCR carried out with the BhRDr-RD5 primers and eight high-quality sequences with the Blast 505-532–Blast 998-1017 primers (Table 2). For seven samples, sequences were obtained with both pairs of primers. According to the best match in terms of BLAST, all sequences were identified as *Blastocystis* sp. ST8 with 97.6–99.8% identity. All sequences were deposited in GenBank (see Supplementary Table for detailed accession numbers). Strain and allele assignment determined using the PubMLST website confirmed matches for ST8 for the barcoding region and the assignment to allele 21, with the exception of two sequences assigned to alleles 156 (OP329405) and 157 (OP329407). The results obtained by ML phylogenetic inferences with the T92+G+I model (G = 0.63 I = 0.56) described the cluster affiliation of the material analysed herein with reference sequences of ST8 available for birds from Japan and captive NHPs for both partial 18S datasets, showing 99% and 100% bootstrap support. The best ML consensus tree obtained from the Santin dataset (primers Blast 505-532 and Blast 998-1017) is shown in Supplementary Figure S1, and the best ML tree from the Scicluna dataset (primers BhRDr-RD5) provided the same topology and is available in Supplementary Figure S2.

**Figure 1:**
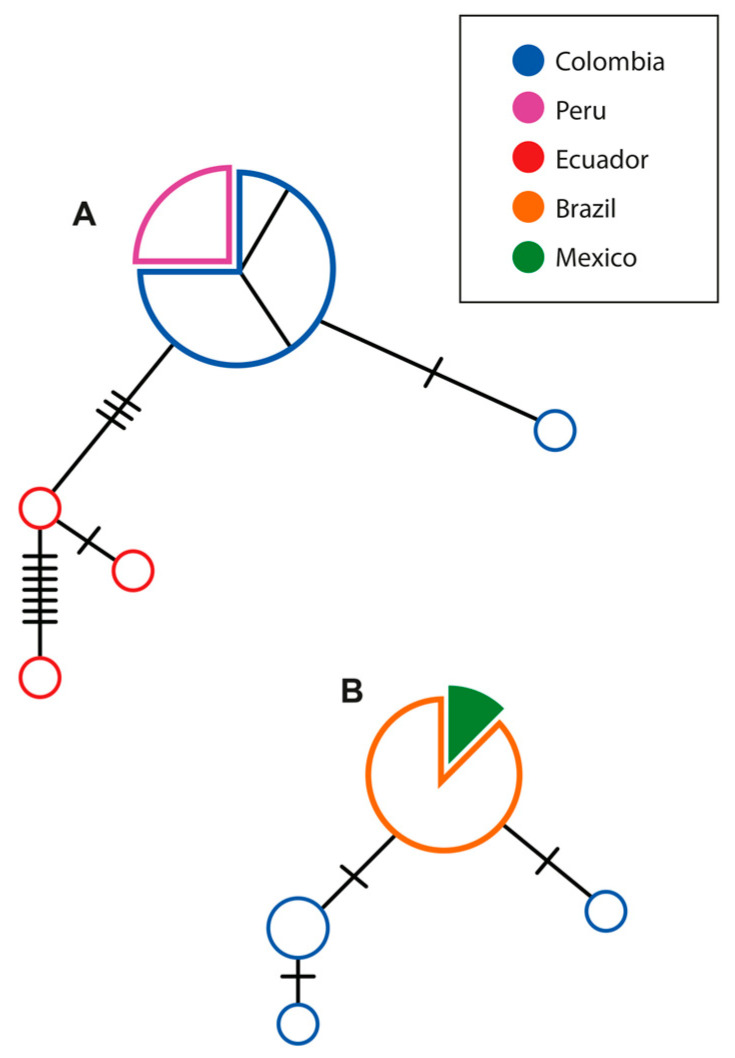
Median Joining Network of *Blastocystis* sp. ST8 partial 18S built with (**A**) the dataset of sequences obtained through the primers by Scicluna et al., 2006 [8] and (**B**) the dataset of sequences obtained with primers by Santin et al., 2011 [26], both circulating in platyrrhines. The size of each circle is proportional to the frequency of the haplotype and the transversal lines indicate an SNP. Internal subdivisions of Colombian samples in (**A**) indicate four specimens from San Juan, four from Yopal, and one from Maní.

The *Blastocystis* sp. ST8 MJ network built with the dataset of sequences obtained with the primers from the research of Scicluna et al., 2006 [8] showed five haplotypes, with a main haplotype shared by most of the sequences of *A. seniculus* from this study (four from San Juan, four from Yopal, and one from Maní) and Peruvian samples of *Aotus nigriceps*, separated by three or more SNPs from haplotypes circulating in *Alouatta palliata equatorialis* in Ecuador. For the dataset of sequences obtained with the primers from the research by Santin et al., 2011 [26], four haplotypes were obtained, each one separated from another by one SNP. Two haplotypes were characterised in the Colombian samples circulating in *A. seniculus* from the Yopal and San Juan regions, which were separated by a central haplotype shared by Brazilian and Mexican specimens reported in several NHP species (*Ateles* sp., *L. lagotricha*, *A. palliata*, *A. pigra*, *A. fusciceps*). Networks are available in Figure 1.

## 4. Discussion

In Latin America, even though Colombia is one of the countries wherein the majority of *Blastocystis* reports originate, surveys including free-ranging NHPs are still very scarce. In this study, we found positive samples for *Blastocystis* ST8, providing new information regarding free-ranging NHPs. Until this study was conducted, in Colombia, *Blastocystis* ST4 was the only identified ST infecting NHPs, which had been reported in two specimens of *Alouatta* sp. [11], while ST8 had only been found circulating in marsupials [11]. Accordingly, this is the first report of *Blastocystis* ST8 infection from Colombian wild NHPs, which was confirmed by phylogenetic analyses.

*Blastocystis* ST8 has already been found circulating in NHPs from South America, particularly in free-ranging mantled howler monkeys from Ecuador [12] and in captive *A. seniculus*, *A. caraya*, *A. fusciceps*, and *L. lagotricha* in Brazil [6]. Moreover, ST8 has been found in captive gibbons and in *Varecia variegata* from a zoo in Spain [4,30]. Human ST8 infections are rarely reported; however, a high prevalence of ST8 has been reported among primate handlers in the United Kingdom [31] and in symptomatic patients in Italy and Australia [32,33]. In Latin America, it has been identified in Brazil in patients with diabetes mellitus and in asymptomatic patients [34,35].

In this study, ST8 allele assignment was prevalently attributed to allele 21, which was previously reported in captive NHPs in Brazil [6] from sequences obtained with the primers used by Scicluna et al. [8], and in Colombia this allele has been found in samples from *Didelphis marsupialis* [11]. Network analyses allowed us to explore *Blastocystis* haplotype lineages and relationships among ST8 strains circulating in primates in Central and South America, for which a generally low level of variation was suggested and only slight differences in the 18S haplotypes were revealed in the Colombian specimens according to the sampling site (San Juan and Yopal). The Peruvian and Colombian sequences appeared to largely overlap, while the samples from Ecuador, Brazil, and Mexico displayed distinct haplotypes.

The analysis based on PCR amplification revealed a lower ability to detect positivity to *Blastocystis* sp. with respect to microscopy. Although unusual, this evidence could be related to factors that may jeopardize the efficiency of molecular characterisation, such as the presence of inhibitors in stool samples, low parasitic loads, and a lack of specificity of the primers employed, in which plant/ant sequences arising from items that are part of the host’s diet are represented.

In this study, ST8 was identified in samples of *A. seniculus*, a species for which a preference for the upper strata of the forest has been observed [36]. A large *Blastocystis* sequence dataset including 30 genera of free-ranging and captive NHPs revealed a cryptic degree of host specificity for some STs; likewise, ST8 was primarily seen in arboreal NPHs native to South America and Asia [4]. Therefore, host specificity, behaviour, and ecological factors may be relevant in shaping the distribution and occurrence of different STs. It is worth underlining that the animals analysed herein did not show any symptoms related to gastrointestinal infections.

The present survey could support the elaboration of a more accurate epidemiological picture of the *Blastocystis* strains infecting NHP species. Surveys including other NHP species are strongly encouraged using both microscopy and molecular analyses, especially for NHP species listed as critically endangered, endangered, or vulnerable according to the IUCN Red List, as in the case of *A. hybridus* and *C. versicolor* included in the present survey. For a better understanding of *Blastocystis* sp. molecular epidemiology, new surveys including humans and other NHP species and aiming to explore the distribution, genetic variation, and host specificity are strongly encouraged.

## Figures and Tables

**Table 1:** Material used for partial 18S sequencing comparison of *Blastocystis* sp. ST8 infecting platyrrhines using Median Joining Network.

Subtype Code	Host	Accession Number	Country
NHPJap1	*Varecia variegata ^ϕ^*	AB107970	Japan
Jap2	Pheasant	AB107971	Japan
AtBr1 a	*Ateles* sp. *^ϕ^*	MG280768	Brazil
LlBr1 a	*Lagothrix lagotricha ^ϕ^*	MG280770	Brazil
ApMe1 a	*Alouatta palliata* ^•^	KT591854	Mexico
ApMe3 a	*Alouatta pigra* ^•^	KT591853	Mexico
AlBr1 a	*Alouatta* sp. *^ϕ^*	MG280771	Brazil
AoBr1 a	*Aotus* sp. *^ϕ^*	MG280767	Brazil
ApEc1 b	*Alouatta palliata aequatorialis* ^•^	KM374608	Ecuador
ApEc2 b	*Alouatta palliata aequatorialis* ^•^	KM374609	Ecuador
ApEc3 b	*Alouatta palliata aequatorialis* ^•^	KM374610	Ecuador
AfBr1 b	*Ateles fusciceps ^ϕ^*	MH784453	Brazil
AnPe1 b	*Aotus nigriceps* ^•^	MT509449	Peru
AnPe2 b	*Aotus nigriceps* ^•^	MT509450	Peru
AnPe3 b	*Aotus nigriceps* ^•^	MT509451	Peru

a: primers—Blast 505-532 and Blast 998-1017 from Santin et al., 2011 [26]; b: primers—BhRDr-RD5 from Scicluna et al., 2006 [8]. ^•^ Free-ranging, *^ϕ^* Captive.

**Table 2:** Prevalence (%) of *Blastocystis* sp. obtained via microscopy, number of positive samples obtained via PCR with each pair of primers, and number of *Blastocystis* sp. sequences obtained per primate species and study site.

			Primers BhRDr-RD5 [8]		Primers Blast 505-532 and Blast 998-1017 [26]	
Study Site	Primate Species	Microscopy *	PCR	Sequencing	PCR	Sequencing
San Juan (06°43′ N 74°09′ W)	*Alouatta seniculus* (n = 28)	21 (75.0%) [55.1–89.3%]	5	5	4	4
	*Cebus versicolor* (n = 20)	6 (30.0%) [11.9–54.3%]	3	0	0	-
	*Ateles hybridus* (n = 13)	7 (53.8%) [25.1–80.8%]	0	0	0	-
	*Aotus griseimembra* (n = 5)	0	0	0	0	-
Cumaral (04°17′ N 73°24′ W)	*Saimiri cassiquiarensis* (n = 42)	11 (26.2%) [13.9–42.0%]	1	0	1	0
Villavicencio (04°06′ N 73°38′ W)	*Saimiri cassiquiarensis* (n = 24)	0	-	-	-	-
Guacavía (04°17′ N 73°30′ W)	*Saimiri cassiquiarensis* (n = 30)	0	-	-	-	-
Cabuyaro (04°17′ N 73°02′ W)	*Sapajus apella* (n = 3)	1 (33.3%) [0.8–90.6%]	1	0	0	
Maní (04°48′ N 72°19′ W)	*Alouatta seniculus* (n = 18)	8 (44.4%) [21.5–69.2%]	1	1	1	0
Yopal (05°16′ N 72°22′ W)	*Sapajus apella* (n = 18)	5 (27.8%) [9.7–53.5%]	5	0	0	-
	*Alouatta seniculus* (n = 11)	5 (50.0%) [18.7–81.3%]	5	4	4	4

* Confidence intervals indicated between the square brackets.

## Data Availability

All data generated during this study are included in this published article.

## Supplementary Materials

The following supporting information can be downloaded at: https://www.mdpi.com/article/10.3390/pathogens12040569/s1, Figure S1: The best ML consensus tree obtained from the Santin dataset (primers Blast 505-532 and Blast 998-1017); Figure S2: The best ML tree from the Scicluna dataset (primers BhRDr-RD5); Table S1: Material used for strain assignment according to *Blastocystis* sp. 18S polymorphisms.
