# Supplementary material for: Prevalence and Molecular Characterisation of Blastocystis sp. Infecting Free-Ranging Primates in Colombia
Source: Pathogens. 2023 Apr 6;12(4):569. doi: 10.3390/pathogens12040569 (PMC10143058; doi:10.3390/pathogens12040569)
Supplement: Supplementary file 1 [file pathogens-12-00569-s001.zip › Table S1.pdf]

Supplementary Table S1. Material used for strain assignment according to *Blastocystis* sp. 18S polymorphisms.

**Subtype code**	**Host**	**Accession number**	**Country**
ST1_USA	Human	U51151	USA
ST1_Thai	Human	AY618266	Thailand
ST2_Jap1	Human	AB070987	Japan
ST2_Jap2	*Macaca fuscata*	AB070997	Japan
ST3_Sen	Human	JX132219	Senegal
ST3_Jap	Cattle	AB107965	Japan
ST4_Ger	Human	AY244620	Germany
ST4_Jap	Rat	AB071000	Japan
ST5_Jap	Cattle	AB107966	Japan
ST5_Ger	Pig	MK801369	Germany
ST6	Human	AB091236	Unknown
ST6_Bra	Chicken	MW538478	Brazil
ST7_Chi	Human	DQ366343	China
ST7_Bra	Chicken	MW538475	Brazil
ST8_Jap1	*Varecia variegata*[ϕ]	AB107970	Japan
ST8_Jap2	Pheasant	AB107971	Japan
512*a	*Alouatta seniculus*[•]	OP328758	Colombia
603*a	*Alouatta seniculus*[•]	OP328759	Colombia
615*a	*Alouatta seniculus*[•]	OP328760	Colombia
616*a	*Alouatta seniculus*[•]	OP328761	Colombia
61*a	*Alouatta seniculus*[•]	OP328762	Colombia
63*a	*Alouatta seniculus*[•]	OP328763	Colombia
79*a	*Alouatta seniculus*[•]	OP328764	Colombia
81*a	*Alouatta seniculus*[•]	OP328765	Colombia
548*b	*Alouatta seniculus*[•]	OP329405	Colombia
603*b	*Alouatta seniculus*[•]	OP329406	Colombia
608*b	*Alouatta seniculus*[•]	OP329407	Colombia
615*b	*Alouatta seniculus*[•]	OP329408	Colombia
616*b	*Alouatta seniculus*[•]	OP329410	Colombia
617*b	*Alouatta seniculus*[•]	OP329409	Colombia
61*b	*Alouatta seniculus*[•]	OP329411	Colombia
63*b	*Alouatta seniculus*[•]	OP329412	Colombia
79*b	*Alouatta seniculus*[•]	OP329413	Colombia
81*b	*Alouatta seniculus*[•]	OP329414	Colombia
ST9_Jap	Human	AF408425	Japan
ST9_Den	Human	KC138681	Denmark
ST10_USA	Cattle	MT898456	USA
ST10_Lib	Dromedary	KC148207	Libya
ST11_USA	Elephant	MT898454	USA
ST12_unk	Wallaby	EU427515	Unknown
ST13_UK	Mousedeer	KC148209	UK
ST14_USA1	Cattle	MT898458	USA
ST14_USA2	Cattle	MT898459	USA

ST15_UK	Gibbon$^{\phi}$	KC148211	UK
ST16_unk1	Red kangaroo	EU427512	Unknown
ST16_unk2	Red kangaroo	EU427514	Unknown
ST17_Lib	Gundi	KC148208	Libya
ST21_USA	White-tailed deer	MW887929	USA
ST23_USA	Cattle	MW887931	USA
ST24_USA	White-tailed deer	MW887928	USA
ST25_USA	Cattle	MW887933	USA
ST26_USA	Cattle	MW887932	USA
ST27_Bra	Indian peafowl	MW887934	Brazil
ST28_Bra	Indian peafowl	MW887935	Brazil
ST29_Bra	Chicken	MW538473	Brazil
ST30_USA	White-tailed deer	MZ267676	USA
ST31_USA	White-tailed deer	MZ267677	USA
ST32_Col	Goat	MZ265403	Colombia
ST33_Col1	Horse	ON932569	Colombia
ST33_Col2	Horse	ON932570	Colombia
ST34_Col	Horse	ON932571	Colombia
ST35_Bra	Human	OP720870	Brazil
ST36_Mex	Bat	OP720871	Mexico
ST37_Mex	Heteromyid	OP720872	Mexico
ST38_UK	Water volpe	OP720869	UK
Proteromonas lacertae		U37108	USA

*samples analysed in the present study; a: primers Blast 505-532 - Blast 998-1017 from Santin et al. 2011[26]; b: primers BhRDr-RD5 from Scicluna et al. 2006 [8].
• Free-ranging, $^{\phi}$Captive.
