# Supplementary figures and images for: Prevalence and Molecular Characterisation of Blastocystis sp. Infecting Free-Ranging Primates in Colombia
Source: Pathogens. 2023 Apr 6;12(4):569. doi: 10.3390/pathogens12040569 (PMC10143058; doi:10.3390/pathogens12040569)

Tree scale: 0.1

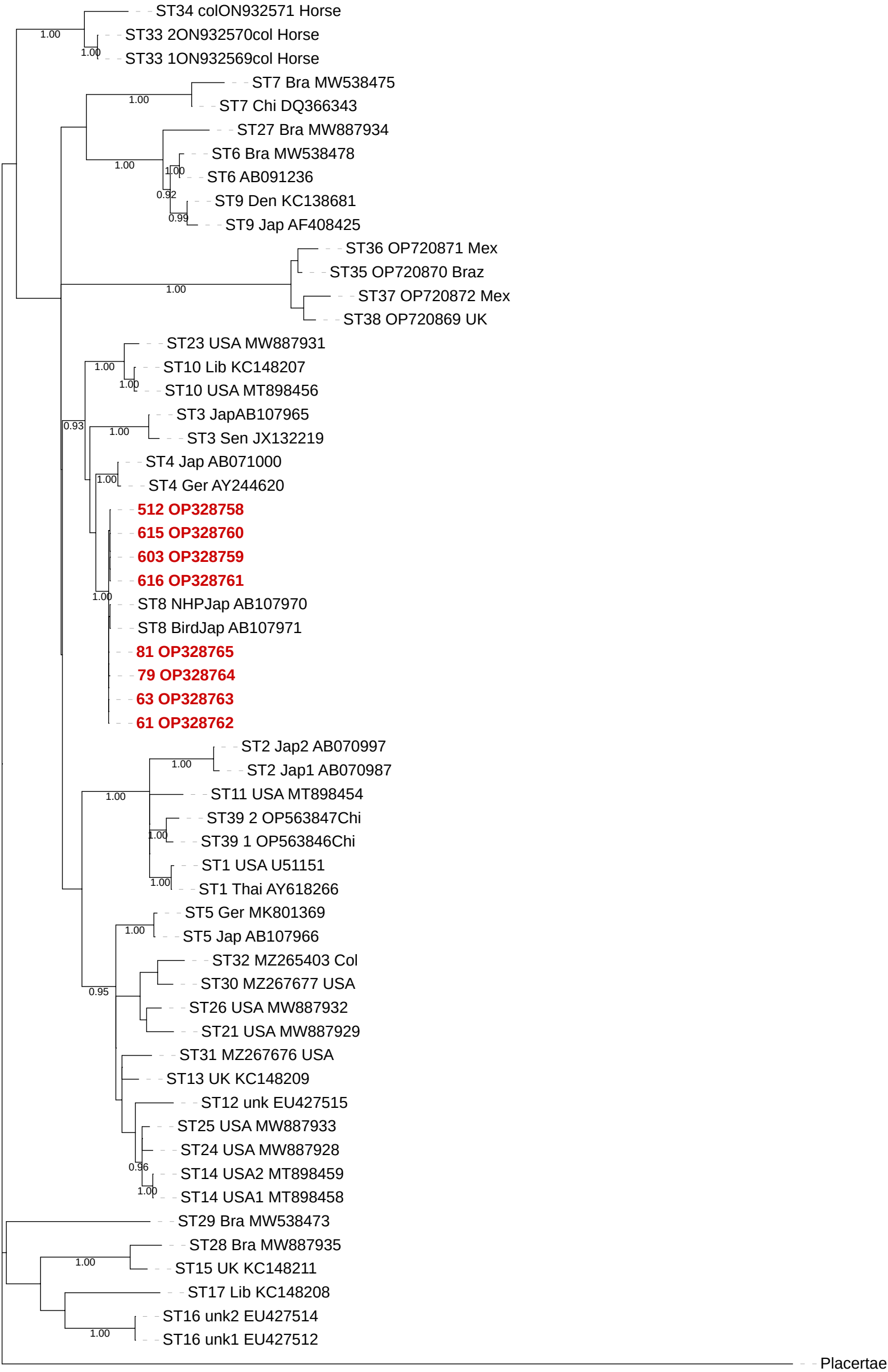

Supplement: Supplementary file 1 [file pathogens-12-00569-s001.zip › Figure S1.pdf]

Tree scale: 0.1

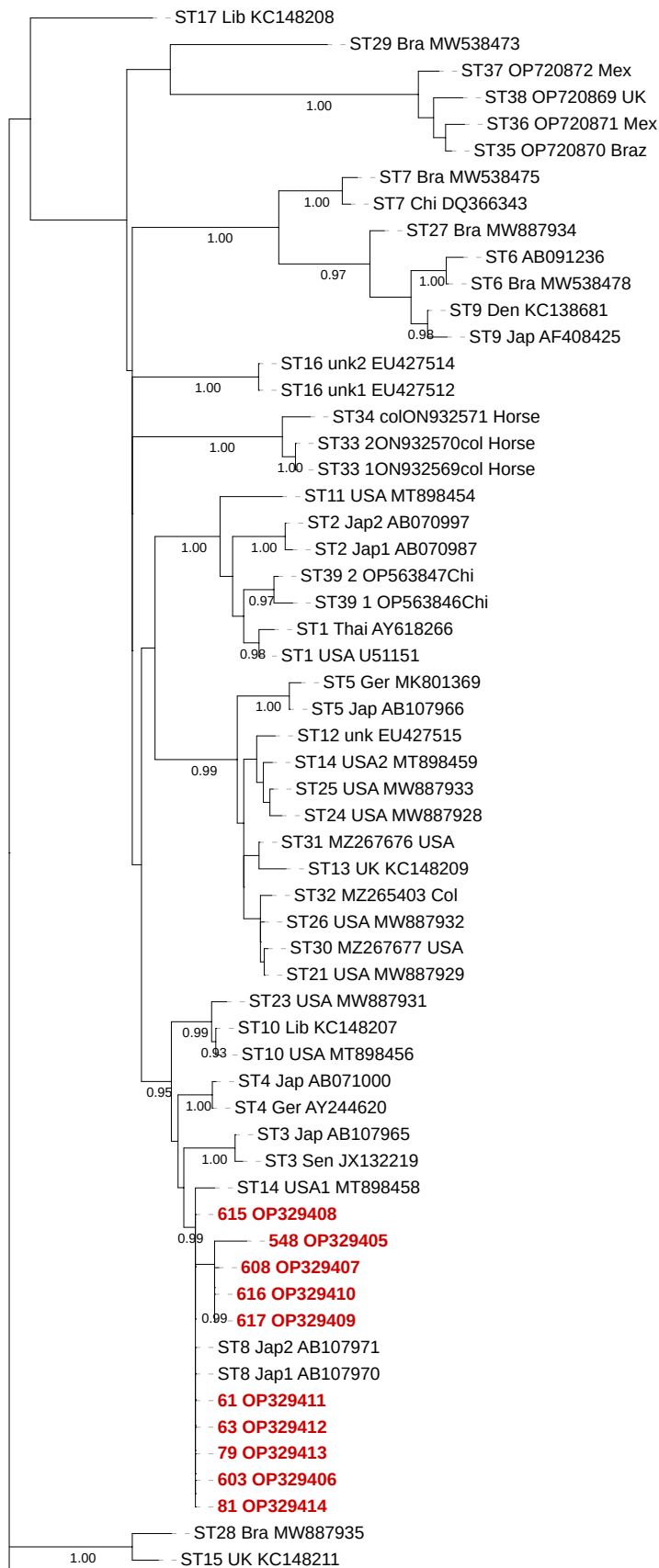

Supplement: Supplementary file 1 [file pathogens-12-00569-s001.zip › Figure S2.pdf]
